# Supplementary material for: The Physico-Chemical Properties of Glipizide: New Findings
Source: Molecules. 2021 May 24;26(11):3142. doi: 10.3390/molecules26113142 (PMC8197375; doi:10.3390/molecules26113142)
Supplement: Supplementary file 1 [file molecules-26-03142-s001.zip › molecules-1222567-supplementary.pdf]

Supplementary material

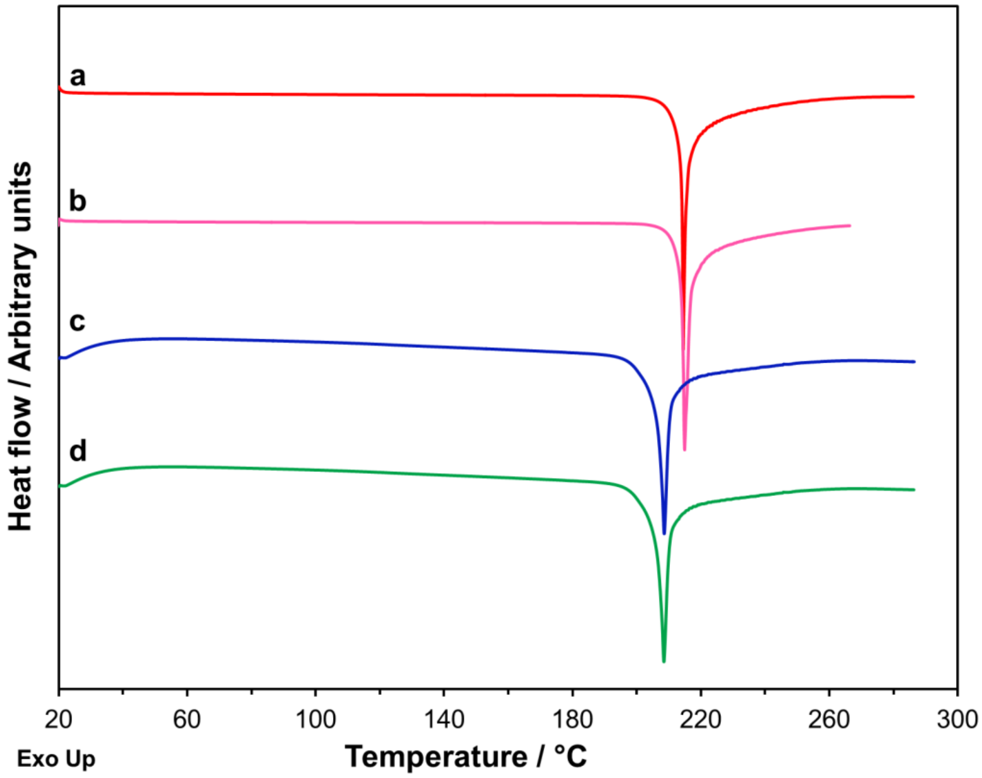

Fig. S1 DSC traces of a production batch (a), USP H1H396 (b), USP R09020 (c) and *GPZ3h* (d) samples at 10 K·min^{-1} heating rate.

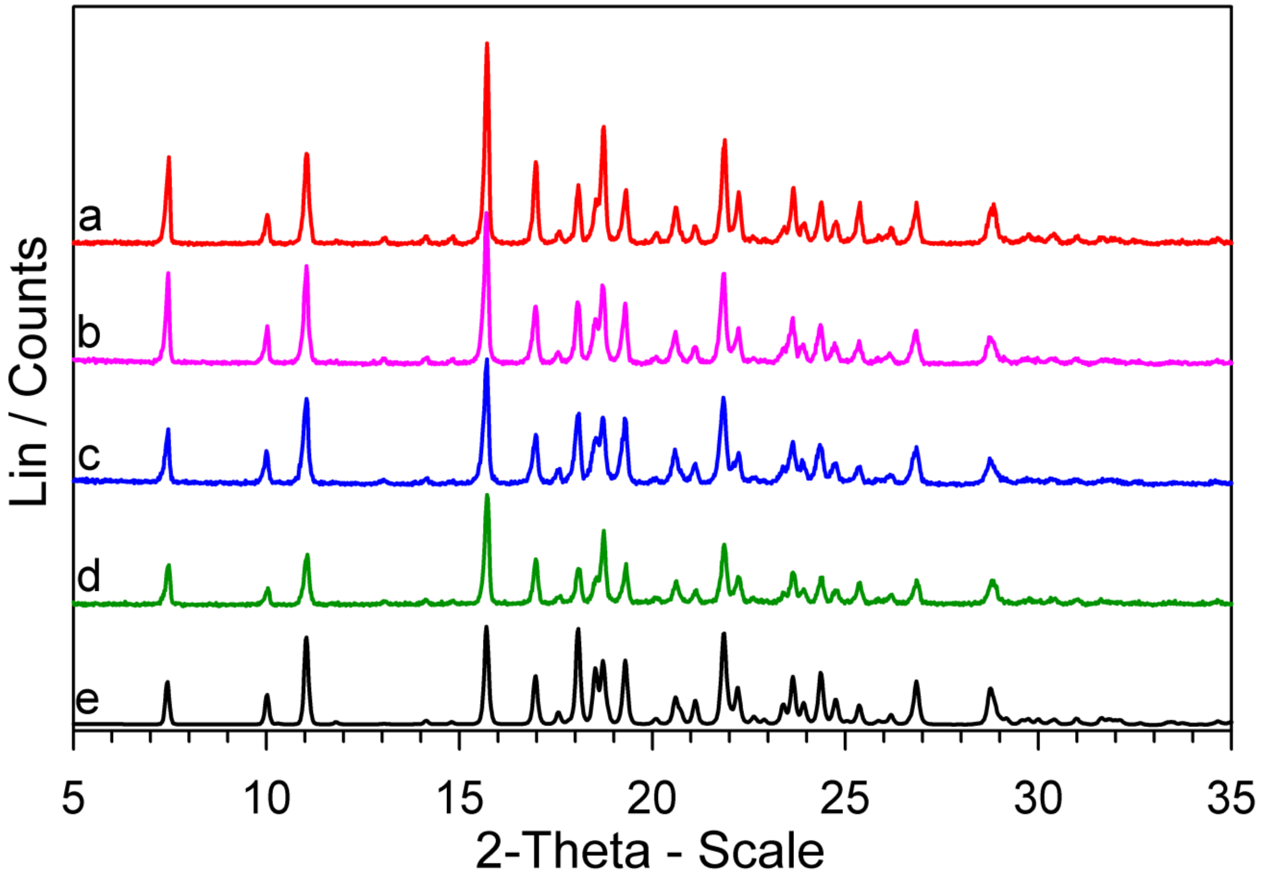

Fig. S2 XRPD patterns of a glipizide production batch (a), USP H1H396 (b), USP R09020 (c) and *GPZ3h* (d) samples. XRPD simulated pattern of GPZ [13] (e).

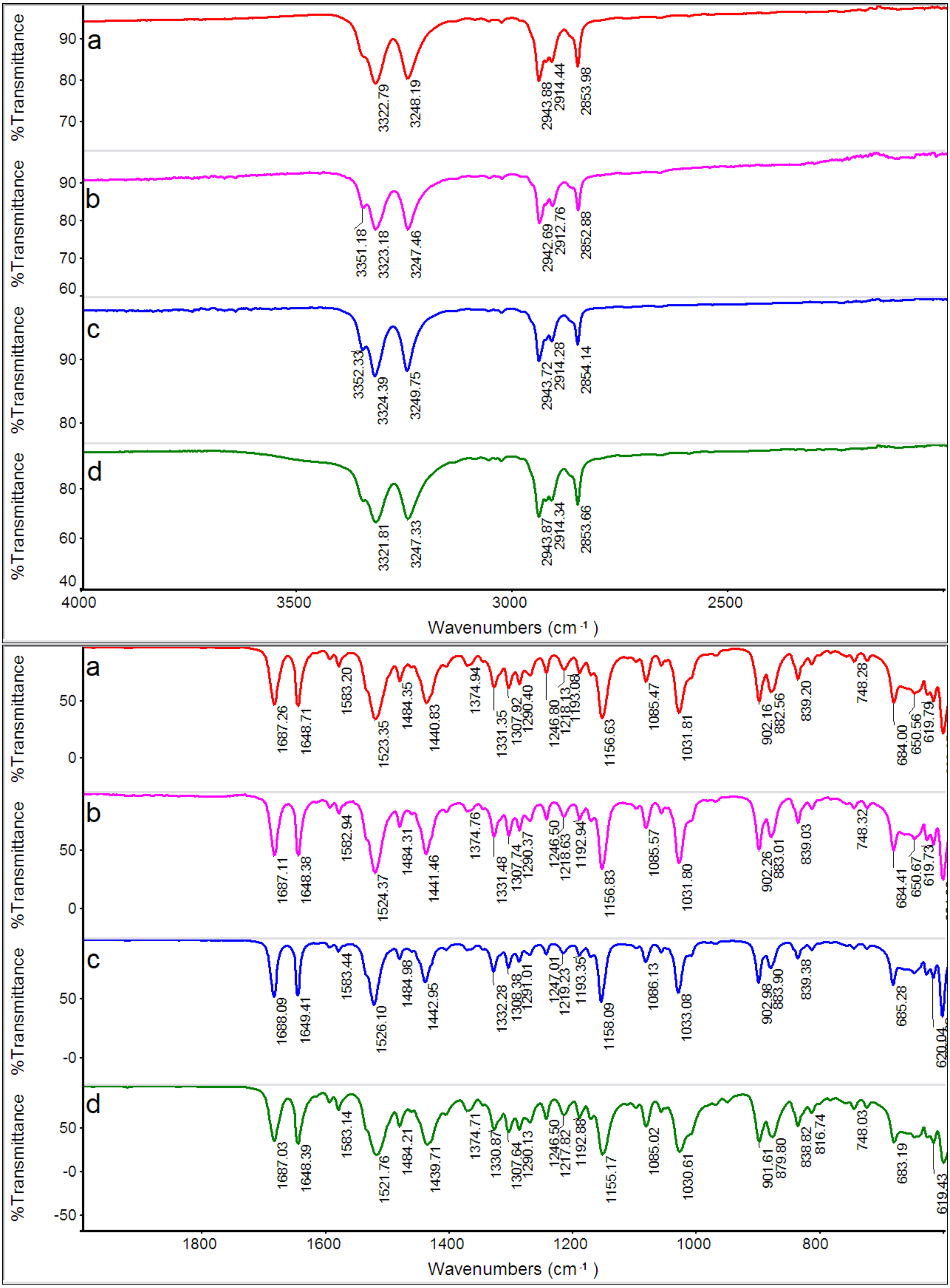

Fig. S3 FT-IR spectra of a glipizide production batch (a), USP H1H396 (b), USP R09020 (c) and *GPZ3h* (d) samples.

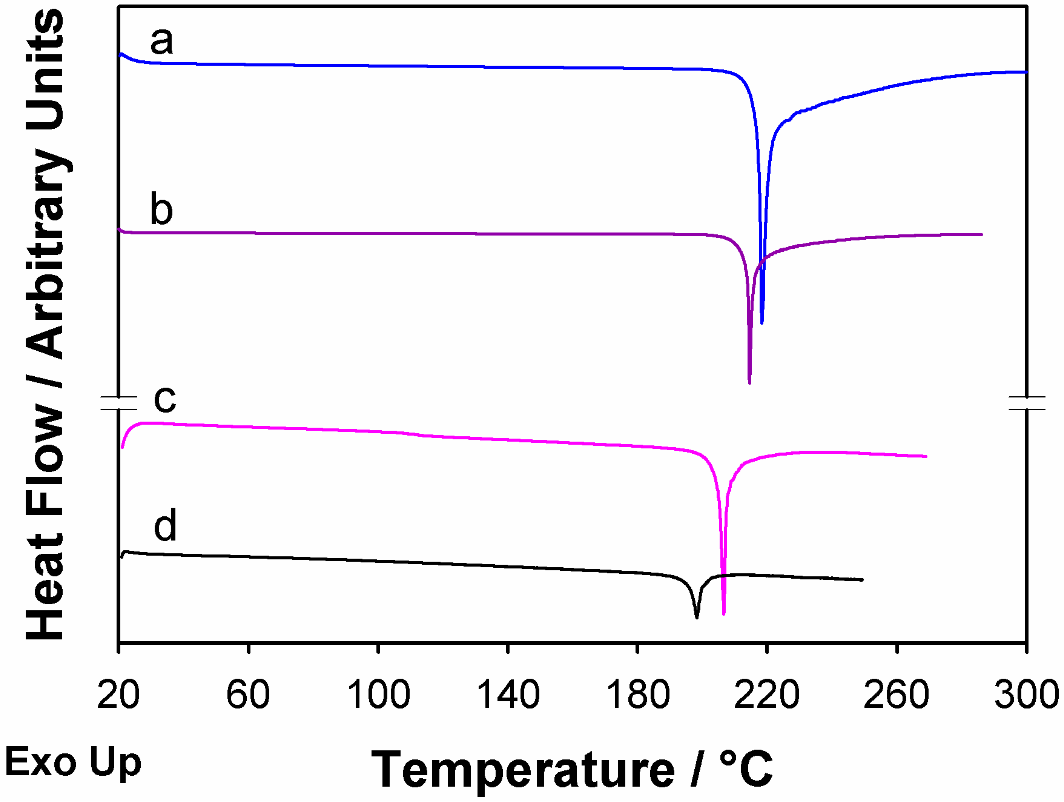

Fig. S4 DSC curves recorded on a glipizide production batch under nitrogen flow at 30 K·min^{-1} (a), 10 K·min^{-1} (b), 2 K min^{-1} (c) and 0.5 K·min^{-1} (d).

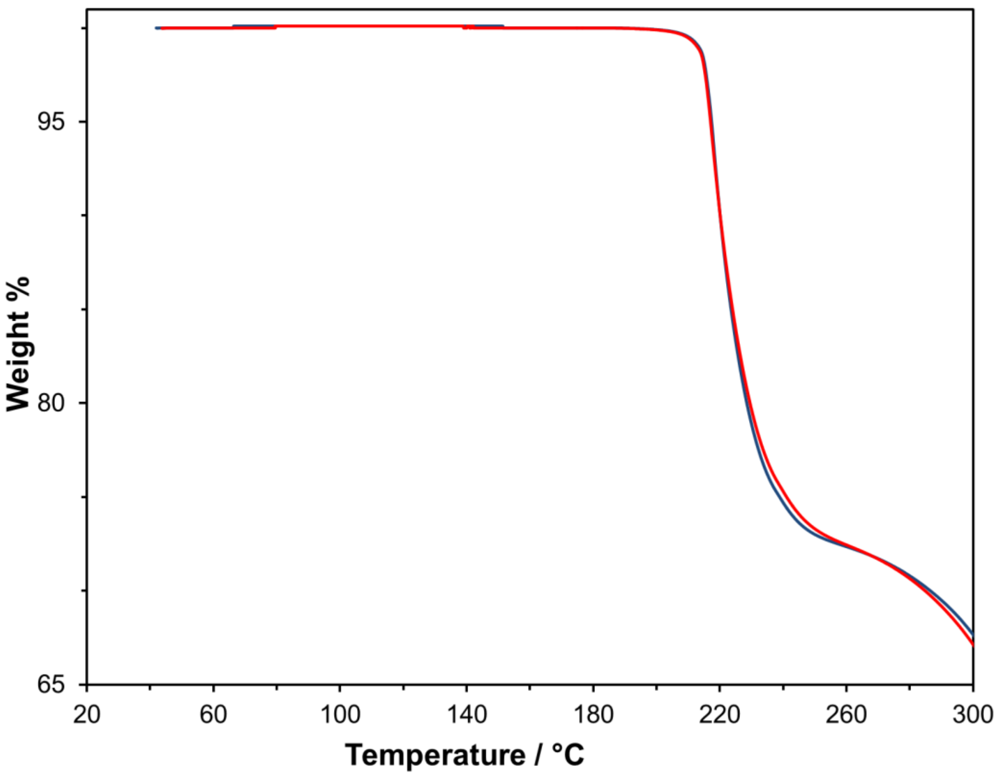

Fig. S5 TG curves registered on a glipizide production batch at 10 K·min^{-1} under nitrogen flow (red) and in air (blue).

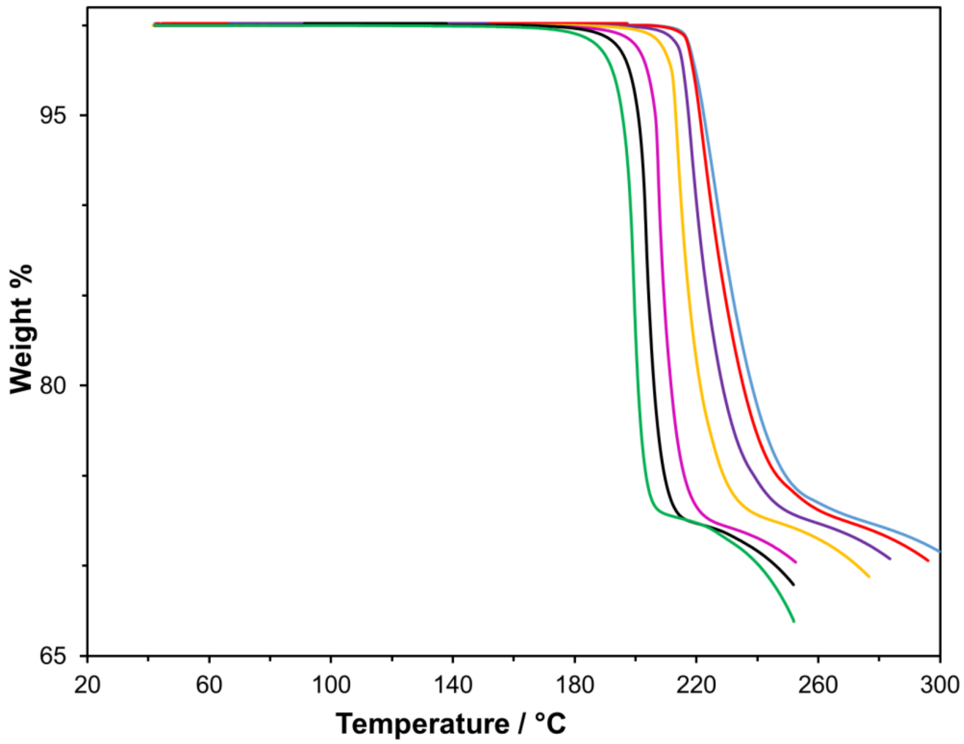

Fig. S6 TG curves registered on a glipizide production batch under nitrogen flow at different heating rates. From right to left: 30 K·min^{-1} (blue), 20 K·min^{-1} (red), 10 K·min^{-1} (violet), 5 K·min^{-1} (yellow), 2 K·min^{-1} (pink), 1 K·min^{-1} (black) and 0.5 K·min^{-1} (green).

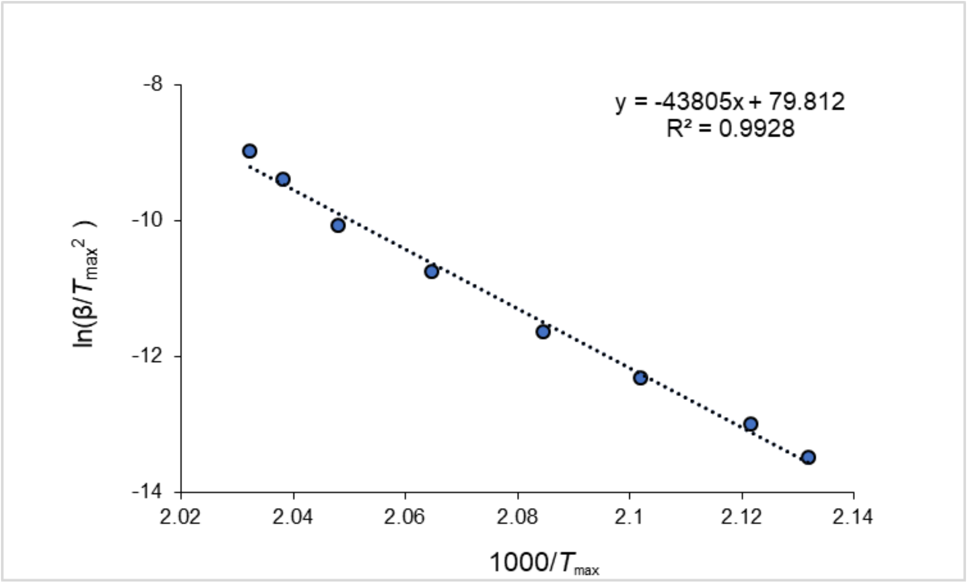

Fig. S7 Experimental data points plotted according to the Kissinger method.

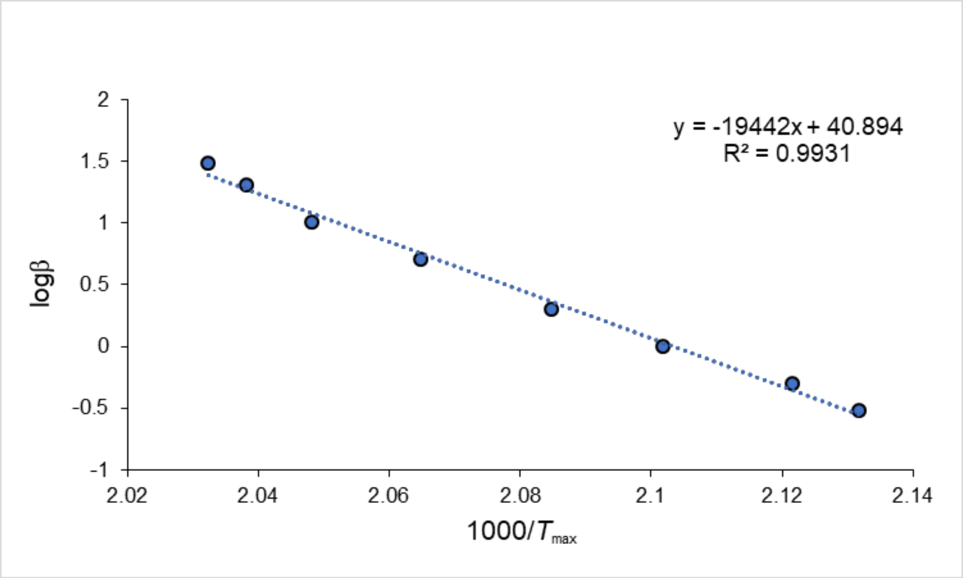

Fig. S8 Experimental data points plotted according to the Ozawa-Flynn-Wall method and their linear regression.

Table S1 Data used to determine the Kissinger equation.

**β, K·min^{-1}**	**T_{max}, K**	**1/T_{max}**	**ln(β/T^2_{max})**
30	492.03	0.002032	-8.9958
20	490.62	0.002038	-9.3956
10	488.23	0.002048	-10.079
5	484.30	0.002065	-10.756
2	479.67	0.002085	-11.653
1	475.75	0.002102	-12.329
0.5	471.34	0.002122	-13.004
0.3	469.06	0.002132	-13.505

Table S2 Data used to determine the Ozawa-Flynn-Wall equation.

**β, K·min^{-1}**	**T_{max}, K**	**1/T_{max}**	**logβ**
30	492.03	0.002032	1.477121
20	490.62	0.002038	1.301029
10	488.23	0.002048	1
5	484.30	0.002065	0.698970
2	479.67	0.002085	0.301029
1	475.75	0.002102	0
0.5	471.34	0.002122	-0.301029
0.3	469.06	0.002132	-0.522878
